# Impact of aging on immunity in the context of COVID-19, HIV, and tuberculosis

**DOI:** 10.3389/fimmu.2023.1146704

**Published:** 2023-05-24

**Authors:** Alba Grifoni, Tonino Alonzi, Galit Alter, Douglas McClain Noonan, Alan L. Landay, Adriana Albini, Delia Goletti

**Affiliations:** ^1^ Center for Infectious Disease and Vaccine Research, La Jolla Institute for Immunology (LJI), La Jolla, CA, United States; ^2^ Translational Research Unit, National Institute for Infectious Diseases “Lazzaro Spallanzani”-IRCCS, Rome, Italy; ^3^ Ragon Institute of Massachusetts General Hospital (MGH), Massachusetts Institute of Technology (MIT), and Harvard, Cambridge, MA, United States; ^4^ Istituto Di Ricovero e Cura a Carattere Scientifico (IRCCS) MultiMedica, Milan, Italy; ^5^ Immunology and General Pathology Laboratory, Department of Biotechnology and Life Sciences, University of Insubria, Varese, Italy; ^6^ Department of Internal Medicine, Rush Medical College, Chicago, IL, United States; ^7^ IRCCS European Institute of Oncology IEO, Milan, Italy

**Keywords:** aging, innate cells, B cells, T cells, COVID-19, tuberculosis, HIV, immunosenescence

## Abstract

Knowledge of aging biology needs to be expanded due to the continuously growing number of elderly people worldwide. Aging induces changes that affect all systems of the body. The risk of cardiovascular disease and cancer increases with age. In particular, the age-induced adaptation of the immune system causes a greater susceptibility to infections and contributes to the inability to control pathogen growth and immune-mediated tissue damage. Since the impact of aging on immune function, is still to be fully elucidated, this review addresses some of the recent understanding of age-related changes affecting key components of immunity. The emphasis is on immunosenescence and inflammaging that are impacted by common infectious diseases that are characterized by a high mortality, and includes COVID-19, HIV and tuberculosis.

## 1 Introduction

**Figure 1:**
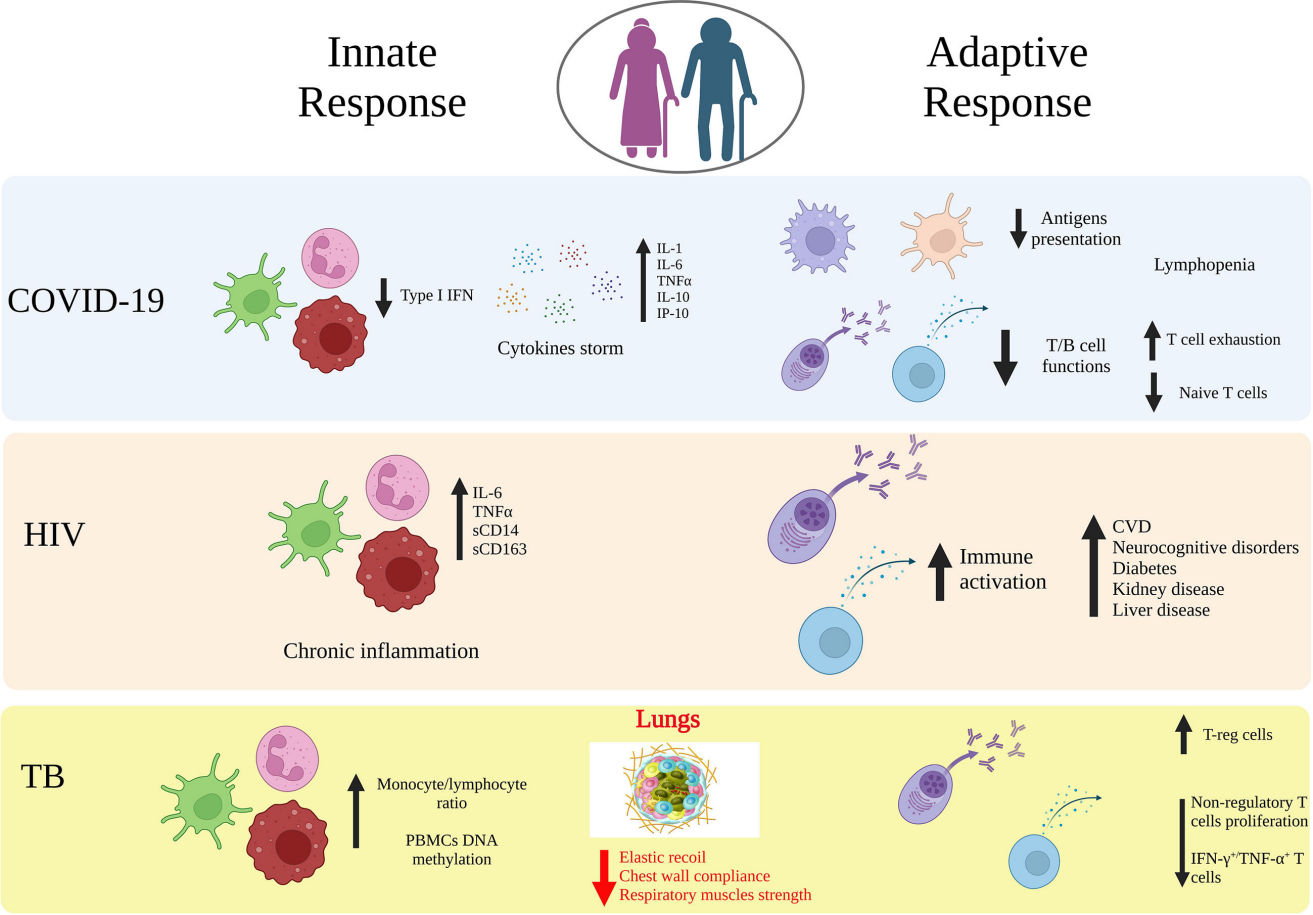
Age-related immune alterations in infectious diseases. Impact of age on the innate and adaptive immunity in COVID-19, HIV and tuberculosis (TB). Aging may lead to modifications of both innate and adaptive immunity arms that induce a dysfunctional immune response against viral or bacterial infections, which includes increased cytokines release, downregulation of APCs, T and B cell functions, upregulation of T-reg cells. Elderly people are an at-risk group for a more aggressive organ damage and the development of secondary diseases. Created with BioRender.com.

It has been estimated that in the next thirty years the number of individuals older than 60 years of age will double, increasing by over a billion individuals; and the number of individuals over age 80 may increase by as much as 300 million people (United Nations Secretariat, E.S.A., 2017). This will lead to a significant increase in age-related diseases. Aging has emerged as one of the greatest and most prevalent risk factors for the development of infectious diseases (1, 2). However, it has been observed that individuals have a different pace of aging which led to the concept of biologic aging. We refer to chronologic aging only with the passage of time, whereas we refer to the biologic aging in relation to functional decline. Biologic aging not only enhances the risk of disease, but further accelerates varied biological processes, which are defined aging hallmarks (3) or pillars (4, 5). Aging impacts nearly all aspects of cellular function, influencing the physiological performance of different organ systems, which induces a decline in immune resilience, with increased susceptibility to infections and mortality. Aging has effects on many cellular processes such as stem cell exhaustion, altered intercellular communication, genetic and epigenetic changes such as DNA methylation, histone modifications, nucleosome positioning, and telomere attrition, decreased capability of protein production and accumulation of misfolded proteins (i.e. loss of proteostasis), dysregulated nutrient sensing, mitochondrial dysfunction, and cellular senescence (5). Cardiovascular disease and cancer are strongly age-associated (3). As we age, the immune system undergoes immunosenescence, which is marked by a systemic process known as inflammaging along with a series of defects in immunological activity that results in poor responses to infectious agents, vaccination and cancer (6–8). Inflammaging is defined as a chronic, sterile, low-grade inflammation that associates with older age, and may contribute to the pathogenesis and clinical manifestations of other age-related diseases (6–8). Inflammaging is due to a loss of control over systemic inflammation leading to a chronic stimulation of innate immunity (7, 9–14). These changes in the immune system, which occur naturally in aging, are linked to an enhanced vulnerability of older adults to disease and death. In particular, aging reduces the ability of the anatomical sites to be barriers to infection, including thinning of the skin, reduced cough reflex, modifications in genitourinary anatomy and bacterial flora and impairment of the bladder capacity and emptying. Comorbidities such as diabetes mellitus and its complications may also weaken host immune defences, increasing risk of infections (i.e foot ulcers, skin and mucosa infections). Neurovascular alterations may enhance the risk of stroke resulting in swallowing dysfunction that may lead to aspiration pneumonia development. Parkinson, Alzheimer, and dementia are a burden of older age. Drug administration can also adversely affect host defence, as well implantable devices, and invasive procedures (15). Relatively healthy, older adults are mostly at risk of respiratory infections (bronchitis, pneumonia) mainly of bacterial origin, followed by urinary tract (in the non-catheterized person) and gastrointestinal infections. Older individuals with specific comorbidities have a higher risk of infection at that organ site (i.e endocarditis with a damaged heart valve, urosepsis with chronic catheter). The risk of infections in the elderly increases in hospitalized patients with aspiration pneumonia, urinary tract infections (for catheter-related issues), intravenous line infections, and infected pressure ulcers (bedridden, immobile). Being older than 85 years of age can be an independent risk factor for admission to the intensive care unit (ICU) and hospital mortality (16). COVID-19, tuberculosis (TB) and Human Immunodeficiency Virus (HIV) infection are the most important infectious disease worldwide accounting for the highest mortality among infectious diseases with more than 1.2 million cases in 2022 for COVID-19 (17–20), 1.6 million estimated deaths for TB in 2021 (21) and 650.000 estimated death for HIV infection in 2021 (22). There were approximately 38.4 million people living with HIV at the end of 2021 with 1.5 million people becoming newly infected with HIV in 2021 globally (22). These diseases have an important health and economic load globally with important consequences in the aging groups. In this review we will focus on immunosenescence during these most common infectious disease (**Figure 1**), highlighting the effect of aging on host innate and adaptive immunity in response to these infections and their contribution on the enhanced disease vulnerability seen in the fragile older adults.

## 2 Aging immunity

### 2.1 Innate immunity in aging

The innate immune system acts as the first line of host defense against pathogens. Innate immune responses limit viral entry, replication, and assembly, identifying and removing infected cells, and coordinating the development of adaptive immunity. Innate immunity involves a broad range of cells, including those from the myeloid lineage: macrophages, monocytes, dendritic cells (DCs), neutrophils and granulocytes, myeloid derived suppressor cells, mast cells, and innate lymphoid cells (ILCs) such as natural killer (NK) cells. Innate immune cells can sense pathogen-associated molecular patterns (PAMPs) or damage-associated molecular patterns (DAMPs) by their pattern recognition receptors (PRRs) to induce inflammatory signaling pathways, followed by the production of inflammatory cytokines and the induction of cell death to clear infected cells (23, 24). Aging of the innate immune system in humans is notable for a paradoxical increase in levels of proinflammatory cytokines, such as interleukin (IL)-6 and tumor necrosis factor (TNF)-α (25). The innate immune system can be trained to respond more rapidly and effectively to infections and this phenomenon is called trained immunity or innate immune memory (26). Trained immunity is characterized by metabolic and epigenetic reprogramming in leukocytes in association with enhanced antimicrobial functions (27). Few studies are available on the effect of trained immunity in aging. Recently, it has been shown that innate immune training can be induced in aging healthy individuals as well as critically ill sepsis patients (28). It can be induced regardless of age and there are not significant differences in the immune trained phenotype as a function of age. Based on the few studies available, trained immunity seems preserved in aged people.

Although the increased inflammation with age is widely discussed, the key innate immune cells believed to facilitate the inflammaging phenomenon are monocytes and macrophages. Several reviews detail how the macrophage and monocyte phenotype and function changes with age (29), as well as neutrophils (30). Differently, how ILCs and NK cells are altered by aging has not been well described in the literature and will be the focus of our discussion below. ILCs represent a relatively recently identified heterogeneous family of mononuclear hematopoietic cells found mostly in solid tissues, which are defined by the expression of factors and cytokines previously associated with T cell lineages. Based on their morphology, gene expression and cytokine production ILCs are classified into three groups ILC1, ILC2, and ILC3. Different than T helper cells, ILCs are activated and regulated by accessory cytokines and germ-line-encoded receptors. Group 1 ILC populations include conventional NK cells and ILC1s and express the transcription factors T-bet or Hobit for their maintenance (31). ILC2s express mainly the transcription factor GATA-binding protein 3 (GATA3) and are associated with type 2 immunity, tissue fibrosis and epithelial repair, dependent on IL-33 (32–34). ILC3s are mainly divided into two subsets, NCR– and NCR+ ILC3 depending on the expression of natural cytotoxicity receptor (NCR) NKp46 or NKp44. ILC3s are grouped into more complex subsets on the basis of their expression of the transcription factors RORγt, RORα, c-Maf, and HIF1 (31). Frequencies and functional potentials of ILC subsets over the human life course have been studied (35). ILCs are more abundant in early life than in later years, but blood CD117^+^ ILC2 are preferentially maintained compared to their CD117^-^ counterparts. ILC2 have been found decreased in aged mice (36), and ILC2-mediated functions repressed in response to aging and high-fat diet (34). ILC3 are important for the activation and activity of NK cells, including cell lysis and cytokine secretion. They produce anti-microbial peptides, IL-17 (important for neutrophil recruitment) and IL-22 (important for tissue repair). They play a role in viral and bacterial infections in the lung and gut (35, 37–39). They decrease with age (35, 38, 40) and this may likely contribute to susceptibility of infections of the elderly population. NK cells are ILCs involved in immunosurveillance by inducing cell lysis both directly (via perforin/granzyme system) or indirectly [via secretion of TNF-α and interferon (IFN)-γ]. One aspect of immunosenescence that has received less attention as compared to other innate immune cells is age-related NK cell dysfunction, characterized by reduced cytokine secretion and decreased target cell cytotoxicity, despite the increase with age (41). Two major human NK cell subsets are defined by the CD56^dim^CD16^+^ and CD56^bright^CD16^-/+^ phenotype. CD56^bright^CD16^+^ are lytic while CD56^bright^CD16^-^ are cytokine producing (41, 42). Elderly individuals have NK cell subsets that are CD56^low^CD16^high^ and CD56^-^, and express check-point inhibitory receptors and activation marker phenotypes (43). Above 50 years of age increasing integrin α4β7 and decreasing C-C chemokine receptor type 7 (CCR7) has been observed (44). Elderly individuals >65 years accumulate the memory-like NK subset CD52^+^ NK2 (NK2.1) that exhibits a type I IFN response and displays proinflammatory characteristics (45). Beside the role that NK cells play against viral infections (33), they are important also in the immunosurveillance as they clear senescent cells, which is linked to age-associated diseases (37, 38). Indeed, senescent cells express NK activating ligands on their surface such as NKG2D ligands, MICA, MICB, CD112 or CD155 which bind to NKG2D receptors or DNAM-1 receptors present on NK cells, respectively, leading to the recognition and killing of senescent cells (46). Senescent cells can upregulate ligands that activate inhibitory signals such as HLA-E or PD-L2, which are ligands for NKG2A or PD-1 receptors expressed on NK cells, respectively (47, 48). NK cell dysfunction is linked to increased incidence of infections, cancer, inflammatory disorders and related to the accumulation of senescent cells with age (41).

In summary, age has an impact not only in the well-studied myeloid cells, but also in innate lymphoid cells and NK cells which are key players against infections. Understanding how NK cells clear senescent cells, as well as how age impacts NK cells is important for increasing our knowledge on immunopathogenesis of aging and for developing targeted therapy.

### 2.2 B cell immunity in aging

Antibodies represent the primary correlate of immunity against infection (49). However, aging is associated with a functional decline in the humoral immune response (50, 51), marked by poor antibody production and the accumulation of aberrant, age-associated B cells (ABCs) (52), compromised germinal center formation (53), and compromised T cell help (54). Specifically, aging is associated with altered responsiveness to vaccines, including reduced influenza vaccine-induced antibody neutralizing titers (51), reduced immunogenicity following COVID-19 vaccination, and reduced durability of vaccine-induced immune responses (55–57). However, in the setting of COVID-19 vaccination (58), the addition of a boost significantly augmented specific immunity in the elderly (59), suggesting that at least some immune defects may be rescued *via* the delivery of additional signals to potentiate the immune response. The induction of potent long-lived vaccine-induced humoral immune responses requires the delicate and coordinated interplay of vaccine antigen uptake by antigen-presenting cells (APCs) that must shuttle antigen to the lymph node and trigger helper-T cells and T-follicular helper cell (Thf) responses, while simultaneously driving antigen deposition on follicular dendritic cells (DCs) that must present antigen to circulating B cells (60). Aging has been associated with significant changes in all cell types involved in the vaccine-induced immune response (61) in terms of phagocytic activity, migratory capacity, inflammatory cytokine release resulting in less potent antigen presentation and priming activity. Interestingly, with aging, T-follicular regulatory cell (Tfr) frequencies decline in the blood and tissues, due to enhanced numbers of Tfr that attenuate Tfh and B cell activation (62). Moreover, as mentioned above, aging is marked by a dramatic shift in the phenotype and function of B cells (52). With aging B cell numbers do not change (63) whereas the bone marrow generates fewer B cells due to alterations in paired box protein (PAX)-5 expression, defects in pro-B cell response to IL-7 (64), and increased TNF-α secretion in the bone marrow, which attenuates the production of immature B cells (63). Instead, ABCs are characterized by distinct phenotypes (CD19^+^, CD21^−^, CD11c^+^, T-bet^+^), gene expression profiles, distinct survival requirements, different B cell repertoire and unique functions. ABCs are not derived from bone marrow B cells but emerge from follicular B cells after extensive affinity maturation, toll like receptor (TLR) stimulation, and proliferation. ABCs are particularly responsive to nucleic acid-sensitive TLRs, including TLR7 and TLR9, independent of B cell receptor stimulation (65). Importantly, these unique properties of ABCs accumulate in autoimmune conditions, and have been shown to contribute to autoantibody production (66). As mentioned above, despite the B cell compartment defects that accumulate with aging, additional boosts as well as novel immunization strategies, including the use of particular adjuvants, have the capacity to rescue these defects and drive robust immunity in the elderly (67). Precisely, against influenza virus, novel adjuvants including MF59 (68) and AS03 (69) have been shown to drive enhanced neutralizing antibody titers in aging populations. Additional boosts have equally been shown to normalize antibody levels to those observed in the general population (59). However, reduced durability of the antibody response remains a challenge, likely requiring additional strategies to promote enhanced T-help to drive longer-lived plasma cell formation.

In summary, antibodies are an important correlate of immunity against infections. Aging is associated with a numerical decline and impairment of humoral response that may limit the ability to contain pathogen growth and response to vaccines.

### 2.3 T cell immunity in aging

T cell responses are a key player of the adaptive immune response to infectious diseases. Adaptive immune responses in the course of a lifespan undergo numerous changes that lead to modifications in T cell subset composition, homeostasis and functionality (i.e. antigen processing and presentation, cell proliferation and activation and signalling pathways) (70). The changes observed in the T cell response can be summarized in the concepts of immunosenescence, inflammaging and enhanced immune suppression. Immunosenescence, defined as the impaired ability to mount immune responses against new infections, is characterized by a reduction of response, which is related to a decrease in the T Cell Receptor (TCR) repertoire diversity and consequent expansion of fewer memory T cell clones (71). As reported above, inflammaging describes the chronic inflammation observed in the elderly and is partially linked to self-reactive T cells inducing chronic tissue damage and low-grade inflammation. Finally, an enhanced immune suppression is observed and linked to an expansion and accumulation of T-reg and Th17 cells in peripheral lymphoid organs with a relative increase in the production of IL-17 and IL-22 cytokines, which are important regulators of proinflammatory cytokines such as IL-6 and TNF-α (72). All those changes in T cell responses are linked to thymic involution, primarily observable during the adolescence phase and characterized by a progressive decline with lymphoid organs being substituted by adipose tissue (8, 73, 74). A secondary atrophy of the thymus occurring around 40-50 years of age, is the one that ultimately reduces the production of naïve T cells (75), although the overall number does not show a comparable drastic decrease due to compensatory mechanisms such as the peripheral clonal expansions of CD4^+^ and CD8^+^ T cells also defined as homeostatic proliferation (76). An additional theory links T cell immunosenescence not only to the thymus but also to the presence of autoantibodies contributing to the deficiency of naïve T cells and increased T-cell activation (77). Therefore, regardless of the cause, with age we observe a progressive decrease of peripheral naïve T cells, in consort with an accumulation of memory T cells with an exhaustion phenotype, loss of the co-stimulatory molecules and acquisition of a differentiated phenotype (78, 79). The change in quality of the T cell responses as a function of age ultimately impairs the ability of T cells to mount a response against new antigens (80, 81). This is particularly evident in the context of CD8^+^T cells where the senescent immune system shows a specific reduction in effector capability, critical for the control of many viral infections (81).

In summary, T cell responses are important in protecting from disease severity. Aging is influencing the naïve T cell repertoire and capability to mount a rapid immune response against a novel viral threat. As we will describe below, this may lead to an increased risk of infectious disease severity as in COVID-19 or to the re-activation of latent infections as observed in the context of TB.

## 3 Specific infection diseases in elderly

### 3.1 COVID-19

Several studies suggest aging as a significant risk factor for severe COVID-19 and increased mortality rate and this is linked to age-related changes observed both in the innate and adaptive responses compartments (82–84) (**Figure 1**). In terms of innate immunity, the cytokines in COVID-19 exert both antiviral and inflammatory activities and promote disease-associated processes directly, such as epithelial cell death and immunothrombosis. Following SARS-CoV-2 exposure, the type I IFN production is increased (85–88). However, patients with severe COVID-19 have altered innate immune function, such as a reduced gene expression (i.e. IFN-β, IL4R; IL10RA, IFNAR1) and activity in response to type I IFN (i.e. TANK binding kinase 1 (TBK1), IFN-regulatory factor (IRF)3, IRF7, signal transducer and activator of transcription 1 (STAT1)/STAT2) (89, 90), thus strongly suggesting that the immunosenescence-associated high viral load could be due to a lower type I IFN response that limits the SARS-CoV-2 clearance. Angiotensin converting enzyme 2 (ACE2) expression levels, the receptor used by SARS-CoV-2 to infect host cells, has been also linked to COVID-19 severity and are affected by age. Counterintuitively, a decline in expression is associated both with age progression and severe disease. This is due to the fact that ACE2 enzyme exerts an innate anti-inflammatory function which is particularly important in the lungs in modulating an excessive inflammatory environment (84). Overall, the impairment of the innate immune response with a combined reduced type I IFN response, and ACE2 activity in the lungs, could contribute to the high levels of pro-inflammatory cytokines and chemokines released, promoting inflammation, vascular permeability, and poor outcomes in older COVID-19 patients (91). To expand more on the levels of pro-inflammatory cytokines, several studies have been shown that SARS-CoV-2 infection triggers in general an excessive induction of multiple cytokines beyond type I IFN, in the lungs and in the blood, which is associated with elevated levels of many pro-inflammatory cytokines, including IL-1, IL-2, IL-6, IL-10, IL-12, TNFα, CXCL10, CCL2, CCL3 (92–95). The levels of these cytokines are correlated with disease severity, and inversely correlated with survival rate. In the general population, inflammaging represents one common feature of biological aging with high circulating levels of pro-inflammatory molecules, such as TNF-α, IL-1, and IL-6 (9). Predisposition to severe symptoms and negative prognosis of COVID-19 has shown to be related with production of cytokines and pro-inflammatory molecules, dysregulation of mammalian target of rapamycin (mTOR), and alteration in the number of innate cells, their activation and polarization (96). Specifically, altered proinflammatory macrophages with consequent recruitment of granulocytes and monocytes in the lung tissue has been reported in severe cases (97, 98). Another hallmark characteristic of disease severity and connected with impaired innate immunity is the decrease of phagocytosis capabilities for neutrophils and macrophages, which is exacerbated by age. NK cell polarization is also affected in severe COVID-19 and age, particularly memory-like NK2.1 cells and related produced cytokines are associated with disease severity in COVID-19 (45). Specifically, IFN-γ due to an early excessive NK cell activation is involved in inflammatory response with a poor prognosis in elderly COVID-19 patients and TGF-β is associated with a shorter hospitalization time of adult patients suggesting a role for TGF-β in preventing an excessive NK cell activation and systemic inflammation (43). Overall, the changes to the innate immune system in older COVID-19 patients can contribute to a greater risk for cytokine storm, due to the proinflammatory environment and reduction in pathogens clearance making them more vulnerable to SARS-CoV-2, and eventually lead to severe cases, more complications, or deaths (10, 11, 99). Humoral responses and particularly neutralizing antibodies are a correlate of protection in COVID-19. The protective immune response against the viral spike protein that prevents SARS-CoV-2 infection has driven the development of vaccines. Four of the vaccines initially authorized for emergency use, Pfizer BNT162b2, Moderna mRNA‐1273, Janssen/J&J Ad26.COV2.S, and Novavax NVX‐CoV2373 COVID‐19 vaccines, were designed based on spike protein, and therefore, most of the knowledge has been obtained about immunity to this viral antigen and, particularly, to its receptor binding domain (RBD) in the context of vaccines. However, there is still a lack of clear definition of a threshold of antibody response sufficient to provide protection against COVID-19 infection or development of severe disease when infection occurs. On the other hand, in several clinical studies of COVID-19 vaccine recipients, both neutralizing and binding antibodies were thought to be highly predictive of immune protection, although this relation is reduced to variants of SARS‐CoV‐2 (100–103). A single dose of mRNA-based vaccine (BNT162b2 or mRNA‐1273) induced IgG and neutralization antibodies, and subsequent doses induced a further increase in both uninfected healthy individuals and naturally infected convalescent individuals. At 6 months post‐vaccination, the antibody activities declined dramatically, and thus additional doses of vaccine are highly recommended to achieve maximum level of protection against COVID-19, especially against the current predominant variant Omicron BA.5 (104–106). Parallel studies have additionally shown that early kinetics of SARS-CoV-2-specific T cell responses and a coordinated adaptive immune response are associated with milder disease (107–110). Taken together, these findings suggest that T cells may act as second line of defense helping in reducing the viral spreading and potentially disease severity. Aging in COVID-19 affects multiple components of the immune system already challenged by the SARS-CoV-2 infection (82, 109, 111). Indeed, the delayed T cell kinetics are exacerbated by an impaired antigen presentation, frequently related to age, but also specifically induced by the SARS-CoV-2 infection (112) as well as a narrow antigen repertoire in contrast to what is observed in milder infection (110, 113). Another hallmark characteristic of the SARS-CoV-2 acute infection is the lymphopenia which has been shown to correlate with increased disease severity and poor outcome. In older patients the lymphopenia is more pronounced as compared to young severe cases, the lower T cell counts further reduce the pools of T cells responding to the infection and limiting its spreading (112). The number of naïve T cells in older adults is decreased, which reduces the development of a robust adaptive antiviral response, thus contributing to the generation of a severe form of COVID-19 (107, 114, 115). Quantity is not the only parameter to consider for T cells, similarly to the humoral response, the maturation status and functionality are also important. In terms of T cell maturation, previous studies have reported a negative correlation between disease severity and frequency of naïve T cells, which is even more pronounced and progressively decreased as a function of age (107, 116). In concomitance with a decline in the naïve repertoire, COVID-19 severe disease is also associated with more terminally differentiated CD8^+^ cytotoxic T cells with reduced functionality (116) and that is particularly common in patients over the age of 80 years (117). Another dysregulation of the T cell response that is common in aging and reported in COVID-19 patients is the T cell exhaustion, particularly for CD8^+^ T cells. However, it is worth noting that many of those conclusions are based on the expression of PD1 and TIM-3 markers (118), associated to T cell activation rather than functional exhaustion during acute viral infections (116, 119, 120). Overall, several reports conclude that a reduction in number and functionality of CD8^+^T cells contribute to severe disease. Conversely, contrasting findings emerge in terms of CD4^+^T cell effector functions where both a decrease in functionality, in terms of cytokine release, and no differences across disease severity have been independently reported (112, 121, 122). Most of the studies reviewed so far, focused on the role of T cell aging in contributing to COVID-19 severe disease. Since the introduction of COVID-19 vaccines, the field is now challenged in understanding how aging is affecting vaccine-induced T cell responses, particularly as age-dependent heterogeneity was observed in the context of the BNT162b2 vaccination (123). Limited studies have been performed so far on the effect of vaccination in elderly with particular emphasis on the T cell response. This remains an important gap of knowledge to address, given the importance of T cell responses in contributing to a protective SARS-CoV-2 specific response, and the resilience observed in cross-recognizing novel variants of concern following vaccination in immune competent (124–128) and immune deregulated individuals as those with multiple sclerosis (129, 130) or immune-mediated inflammatory diseases and/or Rheumatoid Arthritis (131–136). To evaluate potential therapeutic targets against SARS-CoV-2 infection factors influencing immunosenescence and inflammaging processes should be seriously considered. For example, more inflammation caused by innate cell activity has been reported in older males compared to age-matched females, while displaying less adaptive T- and B-cell activities, thus may explaining why older males more likely experienced a severe form of COVID-19 or even death (137, 138).

In summary, aging affects multiple components of the immune system. In the context of innate immunity, there is a direct effect on the viral infection (eg. expression levels of ACE2 that directly modulate infectivity) as well as an indirect effect due to altered functionality of other innate cells such as excessive production of pro-inflammatory cytokines and reduction of phagocytic capabilities directly linked to severe COVID-19. In parallel, adaptive immunity and mainly T cell responses are impacted, providing an additional explanation for the severe symptoms associated with age. Thus, severe COVID-19 is due to multifactorial immune components independently affected by age, highlighting geroscience’s importance in thoroughly dissecting all the pathogenetic mechanisms and providing better vaccine strategies and therapeutic approaches in the elderly population.

### 3.2 HIV

The field of HIV research has substantially advanced over the past forty years based on the introduction of highly active antiretroviral therapy. This has led to persons who are living with HIV (PLWH) to live to older ages even approaching normal life span (139). There exists however the potential that HIV infection may lead to accelerated or accentuated aging with the development of non-communicable diseases at an earlier age (140). This includes the development of cardiovascular, metabolic diabetes, neurocognitive, bone, liver and kidney disease (141–144). The underlying driver for developing these non-communicable diseases is a subject of active investigation. One of the major areas of study here is persistent inflammation in PLWH that has been recognized as a major contributor to these conditions (145). The main contributor to this inflammation appears to be the activated innate immune system (**Figure 1**). The markers of inflammation include finding persistent elevation of IL-6 and TNF-α as well as C reactive protein and D-dimer. The role of microbial translocation and the gut microbiome have received significant attention in contributing to the persistence of these inflammatory markers. The biomarkers of gut translocation include intestinal fatty acid binding protein, zonulin, lipopolysaccharide binding protein (LBP), and beta-D-Glucan. The bacterial translocation marker LBP (146) and the fungal biomarker beta-D-Glucan (147) have been shown to stimulate innate immune effector cells including monocyte/macrophages that leads to elevated levels of inflammatory mediators and drive the development of the non-communicable diseases in the aging HIV population (148). From the perspective of the microbiome changes in HIV-infected patients and aging we recognize the critical role of microbial metabolomic products. The most relevant of these are the short chain fatty acids which are reduced in PLWH and the loss of these metabolites has been shown to contribute to the persistent activation of innate immune effector cells and non-communicable diseases. We also recognize that other co-infections in PLWH may contribute to aging and these include Cytomegalovirus (CMV), Hepatitis B Virus (HBV) and Hepatitis C Virus (HCV) infections. The role of CMV in aging and on the immune system is especially critical as this virus can contribute significantly to T cell senescence (149). On a global basis other prominent infectious disease such as tuberculosis and arbovirus infections (i.e. dengue) found especially in resource limited settings, can be an important driver of inflammation and co-morbidities such as cardiovascular disease. Other critical contributors to inflammation include life style factors such as smoking, alcohol and diet. We recognize especially the critical interactions of alcohol with the immune system and the gastrointestinal tract that can contribute to microbial translocation and reductions in short chain fatty acid producing bacteria. The study of HIV disease has now become much more integrated into the aging biology field with the geroscience movement. The concepts of inflammaging and senescence-associated secretory phenotype (SASP) are being evaluated more and more in PLWH. We are working to move more concepts of gerontology into the HIV field including the evaluation of frailty as a critical way to focus on the functional assessment of the whole person (150). The features of both innate and adaptive immunity are now well integrated into our thinking of HIV pathogenesis and aging.

In summary, HIV can be considered a disease of accelerated and accentuated aging linked to multiple non-communicable disease outcomes. The cause of this aging phenotype in HIV is multifactorial and includes inflammaging, microbial translocation, coinfections, and life style factors. The study of HIV and aging now intersects much more with applications of geroscience providing novel insights into the pathogenesis of HIV.

### 3.3 Tuberculosis

It is known that aging is a major risk factor for developing TB disease (151). Although, TB notification rate decreased in the period 2016-2021, likely due to the COVID-19 pandemic (17–19), 10.6 million are the estimated new cases worldwide in 2021 and the elderly (>65 years) account for almost 1,400,000 cases (about 13% over total) (152). Elderly population is at higher risk for both the reactivation of TB infection and for new Mycobacterium tuberculosis (Mtb) infections, with higher mortality rates and adverse drug reactions (153–157). In addition to the presence of co-morbidities as diabetes (158) and concomitant drug use with decreased renal and hepatic drug clearance (159, 160), aging is associated with a progressive reduction in the respiratory system function. At an anatomical structural level, the lungs have a decreased elastic recoil, reduced chest wall compliance and show weakness of the respiratory muscles (161), thus compromising the efficacy of the airways secretion clearance. Although it is known that immune-senescence and inflammaging are natural processes that occur in both circulating cells and tissue immune cells, the influence of age on the interplay between these cells during Mtb infection is not understood and it is understudied. The host-Mtb interactions are complex and only partially explained. When Mtb invades a susceptible host, neutrophils, monocytes, DCs are infected in the lung and then an innate granuloma is formed. Then, T-cell specific response is generated in the draining lymph nodes and they reach the granuloma (162, 163). It has been demonstrated that the lungs of old mice have elevated levels of proinflammatory and Th1 cytokines (TNF-α, IFN-γ, IL-12) and the resident macrophages express elevated levels of IFN-γ-induced activation markers (IRGM-1, IRF-1, CIITA). Notably, those macrophages infected *in vitro* have increased Mtb uptake and phago-lysosomal (P-L) fusion but a defect in intracellular control of mycobacterial growth. These data indicate that age induces an inflammatory pulmonary environment which makes the resident macrophages more prone to be infected by Mtb, at least in mice (164). This is partially in agreement with the known altered monocyte proportion (increased monocyte/lymphocyte ratio) (165, 166) (**Figure 1**). T cells are crucial players in the host defence and in containing the spread of Mtb during acute infection and reactivation. T lymphocytes subsets are pivotal for the regulation of the immune response as demonstrated by the lack of Mtb containment during HIV-coinfection and increased HIV replication in coinfected patients (167–169) or by the impairment in coinfection TB-COVID-19 (170–173). It is reported that an imbalance between Th1- and Th2-like cells plays an important role in disease outcome in human TB, with a Th2 polarized response as the major contributing factor for immunosuppression and dissemination of the disease (169) whereas a Th1 polarized response is associated to cell–mediated host antimycobacterial defence (174). Conflicting results on the Mtb-T-cell-specific response of circulating cells in the elderly population are available. It has been reported that the IFN-γ production measured by IFN-γ release assays (IGRA) (175) is either negatively correlated with age (176) or not changed (166). It has been speculated that the PD-1/PD-L1 pathway impairs Th1 immune response in the late stage of infection (177). Interestingly, a trend for increased PD-1^+^ cells in both CD4^+^ and CD8^+^ lymphocytes in elderly patients with TB disease has been observed (166). However, the regulation of the Th1/Th2 balance is not sufficient to control Mtb replication (178). Other T cells such as T-reg cells are fundamental in the immune response against Mtb (179, 180). The frequency of circulating T-reg cells (CD4^+^ CD25^+^ FoxP3^+^) is higher in older TB patients as compared to younger counterparts (181). Interestingly, these cells have an increased proliferative rate in response to Mtb antigen stimulation *in vitro*, with the concomitant reduction of the proliferation of non-regulatory cells (CD4^+^ CD25^−^ CD127^+^ FoxP3^−^ Ki67^+^). Notably, the depletion of the T-reg population resulted in the rescue of effector T-cell functions as measured by the frequencies of IFN-γ^+^, TNF-α^+^ single, as well as polyfunctional (IFN-γ^+^ and TNF-α^+^) T cells (181). It has to be considered that differences in the memory immune cells repertoire may exist among the different anatomical sites, for instance peripheral blood vs tissue. Aged individuals with detectable circulating T cells responding to Mtb antigens were nonresponsive locally as evaluated by skin tests (182). Senescent or exhausted T cells display molecular signatures of aging, such as mitochondrial dysfunction and epigenetic remodelling (183). Interestingly, it has been reported that patients with TB have DNA hypermethylation of multiple genes of the IL-12/IFN-γ signalling pathway, associated with decreased immune responsiveness, IFN-γ–induced gene expression and decreased IL-12–inducible upregulation of IFN-γ (184). Moreover, it has been reported that peculiar DNA methylation signatures may influence individuals prone to be infected by Mtb (185). Overall, evidence strongly indicates that epigenetic modifications of immune cells of TB patients, in general and more specifically in the elderly, are critical for Mtb immune response. Human and non-human primates have unique Vγ2Vδ2 T cells, which are the sole γδ T-cell subpopulation capable of recognizing the microbial (E)-4-hydroxy-3-methyl-but-2-enyl pyrophosphate (HMBPP) produced by selected pathogens such as Mtb (186). Interestingly, it has been reported that aging induces a shift in the central and effector memory populations from Vγ9Vδ2 to Vγ2Vδ1, thus indicating that this T cell population may be involved in the higher susceptibility to TB of elderly people (187). Overall, the few data available on the aged TB patients show that aging is an important determinant for the quality and magnitude of systemic inflammatory and specific immune response to Mtb. Since it has been shown that in old people an elevated number of T-reg cells correlates with bacillary load and disease severity, it may be plausible that changes in the immune system related to senescence may contribute to the increased risk of either new infections or Mtb reactivation in the already infected people (181).

In summary although almost 13% of TB patients are over the age of 65 (21) accounting for 1.4 million individuals, little research has been performed on this population. In the future for clinical and immunological research topics, it will be important to better understand in the elderly the immune mechanisms that may lead to TB progression versus Mtb containment. Since the average life span of the worlds’ population is constantly growing, progress towards the development of an effective vaccine and/or the development of adequate therapy for aged TB-infected people is needed. It is likely that a multidisciplinary approach involving infectious disease specialists and geriatricians will be required to improve clinical outcomes of Mtb infection in this vulnerable population. In fact, the Mtb-induced morbidity is not due exclusively to the pathogen loads but also to the capability of the host to resolve the inflammatory reaction.

## 4 Possibilities of strengthening immunity in old age

Aging is a very complex process which involves both biological features and environmental and psychological aspects. To improve immunity in the elderly it is, therefore, not sufficient to have treatments aimed at increasing the innate or specific immune response against microorganisms, it is also necessary to lower the inflammaging process. In other words, a holistic approach, which involves specific treatments, nutrition and life style is necessary to achieve this goal. Several approaches can be proposed to strengthen immunity against infections in old age, which includes vaccination, modulating inflammation and microbiome composition. Vaccinations are recommended in the elderly, with the purpose to generate specific immunity that reduces infections, disease severity and mortality. Recommendations are mainly for vaccinations against Influenza (188), SARS-CoV-2 (189), Pneumococcus (190) and Herpes Zoster (191). Vaccination against tuberculosis is not effective in adults or in the elderly to strengthen tuberculosis-specific immunity (192, 193). On the other hand, non-specific effects of vaccination have been reported (194) and may represent a way of overcoming immunosenescence in elderly people. Bacillus Calmette Guerin (BCG) vaccination is one of the most studied. It has been reported that BCG vaccination decreases the inflammatory state of elderly people, decreasing the plasma levels of proinflammatory cytokines (i.e. TNF-α, IL-6, and IL-1β), chemokines (i.e. CCL2 and CXCL10), matrix metalloproteinases, acute phase proteins (including C-Reactive Protein) one month after inoculation (195). Conflicting results have been reported on the role of BCG vaccination to protect elderly people from lower respiratory tract infections of viral origin including COVID-19 (196–198). It is however important to highlight that the BCG-vaccination improved the cytokine response and the production of antibody titers after COVID-19 infection (195, 197). In another trial, BCG vaccination decreased the risk of COVID-19 at six months post-vaccination while this approach failed at three months post-vaccination (175). Using a different vaccination strategy, live-attenuated Varicella Zoster (LAVZ) vaccine was shown to decrease the likelihood of being infected by SARS-CoV-2 in old people (199). The prophylactic function of BCG, LAVZ or other vaccinations has to be fully established; however, they may likely be used as a potential adjuvant to mitigate the effects of concurrent respiratory infections. Trained immunity has been hypothesised as a possible mechanism of action probably through the metabolic and epigenetic modifications induced by vaccination, as described for BCG (200). Since aging affects many intracellular processes, one possible strategy to improve immune cell fitness could be to provide elderly people with cellular supplement compounds such as antioxidants or nutrients. One example has been reported in HIV-infected individuals in which glutathione treatment for 13 weeks improved the levels of Th1-type cytokines circulating levels and *in vitro* reduced the intracellular survival of Mtb in infected PBMCs (201). Notably, supplementing aged people (61–80 years old) with a combination of the glutathione precursor amino acids glycine and cysteine (GlyNAC= glycine and N-acetylcysteine) for 16 weeks improved the oxidant damage, mitochondrial dysfunction, inflammation and endothelial dysfunction (202). A randomized case-controlled trial demonstrated that a supplement containing Sambucus nigra, zinc, tyndallized Lactobacillus acidophilus (HA122), vitamins (C, D, E) arabinogalactans, did improve both the inflammatory state and lymphocyte growth of people aged over 60 years (203). In line with these data are results of several clinical trials of elderly people receiving vitamin D supplementation that ameliorated the clinical score, the need for oxygen and intensive care support and survival rates (204). Along with these strategies, the microbiome, the community of microorganisms (i.e. fungi, bacteria, protozoa and viruses) present in the human body, can play a large part in inflammaging, especially when infectious diseases exert changes in the gut microbiome environment. The microbiome has a fundamental role in the development of the immune system throughout the lifespan of an individual. In advanced age, there are several aging-related changes that occur within the gut, including decreased epithelial cell proliferation, stem cell retention, and barrier function, as well as increased gut leakiness and proinflammatory dysbiosis (205). Aging-related changes to the microbiome can lead to changes in overall health of an individual, leading to further increases in inflammaging, and a proinflammatory state (206). There may be ways in which one could attempt to address or prevent aging-related deterioration of the microbiome. These approaches may include prebiotic supplementation which may be in conjunction with postbiotics to target an increase in beneficial microbial taxa and a reduction in taxa related to unhealthy aging (207, 208). Research is ongoing as to whether these types of interventions are beneficial or even functional in humans. Pilot studies of polyphenol-rich foods, prebiotics, and some probiotics have demonstrated some efficacy in older individuals with comorbidities (frailty, insulin resistance, obesity) (209). Interestingly, the use of probiotics as an adjuvant for influenza vaccination in a placebo-controlled, randomized, double-blind, clinical trial was found to be effective in reducing common infectious diseases symptoms and incidence (210). Other approaches to address inflammaging include the use of metformin or IL-6 inhibitors to reduce overall inflammation in the aging population. Metformin is widely used in the treatment of type 2 diabetes, and it may also promote healthy aging through a yet unknown mechanism, including potentially promoting a healthy microbiome in the gut (211). Efficacy of metformin may vary on an individual basis. As described above, the dysregulation of cytokines is implicated in inflammaging and in particular IL-6 is associated with increased age-related comorbidities, including frailty, risk for cardiovascular disease, diabetes, muscle loss, functional decline, and mortality (212–214). Therefore studies to evaluate the effect of IL-6 inhibitors in the aging populations may be beneficial to assess their role in preventing unhealthy aging. Further research is needed to conclusively determine interventions that will address aging-related changes to the microbiome that may lead to decreased risk of developing age-related systemic comorbidities.

In summary, there are a number of possible therapeutic areas including vaccination, inflammation modulators, vitamins, probiotics. Further studies are needed to identify those that may be pursued in ameliorating the impact of aging on infectious disease.

## 5 Conclusions

There is a strong link between the biological hallmarks of aging and the increased susceptibility to severe disease outcomes seen with biological aging. New research that may lead to the discovery of distinctive “biological hallmarks of aging” responsible for the immune deterioration and diminished resilience is often associated with aging (5). Novel therapeutic approaches need to be generated in order to improve infectious disease outcomes and enhance health in older adults.

## Author contributions

DG, AG, AA, AL, GA: Conceptualization. AG, TA, DN, GA, AL searched literature. AG, TA, DG, AA, AL, GA wrote first draft. AG, AA, TA, GA, DN, DG wrote and edited overall. All authors contributed to the article and approved the submitted version.
